# Bidirectional Circumferential Intimo-Intimal Intussusception in Acute Aortic Dissection

**DOI:** 10.1016/j.atssr.2023.05.010

**Published:** 2023-06-07

**Authors:** Emily Rodriguez, Kelly Ohlrich, Michael Robich, Eric Roselli

**Affiliations:** 1 Division of Cardiac Surgery, Johns Hopkins Hospital, Baltimore, Maryland; 2 Division of Cardiothoracic Surgery, Medical University of South Carolina, Charleston, South Carolina; 3 Department of Thoracic and Cardiovascular Surgery, Cleveland Clinic Foundation, Cleveland, Ohio

## Abstract

Complete circumferential aortic dissection with bidirectional intimo-intimal intussusception is a rare occurrence in Stanford type A dissections. The antegrade dissection flap can obstruct the left ventricular outflow tract and coronary sinuses, whereas the retrograde flap can obstruct the aortic arch and branch vessels. Sequelae include aortic regurgitation, myocardial ischemia, and neurologic complications. This case series highlights the need for prompt diagnosis with electrocardiography-gated computed tomography and surgical intervention for this unique pathologic process.

A complete circumferential tear of the intima with a proximal intimal flap obstructing the left ventricular outflow tract (LVOT) is a rare and potentially lethal occurrence in Stanford type A aortic dissection (AD).^1^ Such a pathologic process is referred to as aortic intimo-intimal intussusception (AoII; Figure 1). Exceedingly rare are cases of bidirectional AoII, in which the proximal/antegrade and distal/retrograde tears obstruct both the LVOT and aortic arch, respectively. We report a case series of 3 patients who presented with bidirectional circumferential AoII in Stanford type A AD.

**Figure 1:**
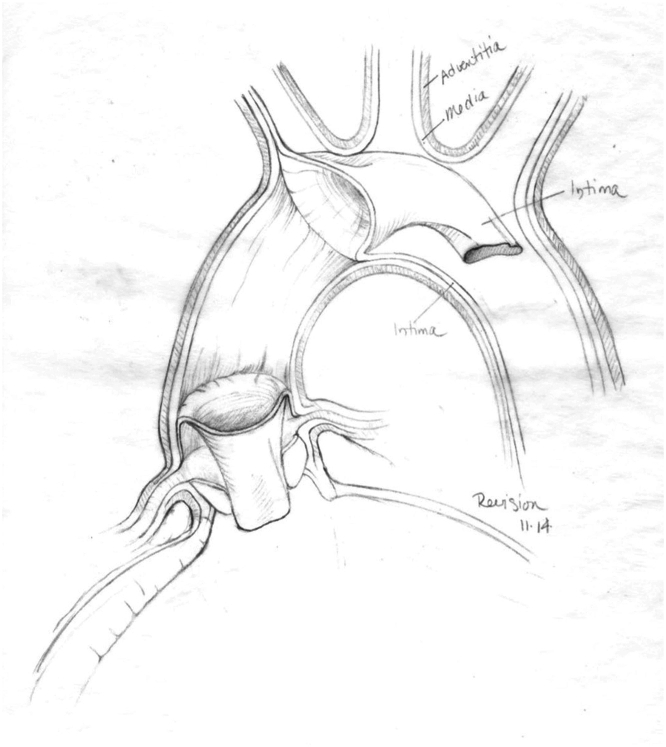
Sketch of aortic intimo-intimal intussusception with dissection flaps protruding antegrade into the left ventricular outflow tract and retrograde past the aortic arch branch vessels.

## Case Reports

### Patient 1

A 47-year-old man with no significant past medical history presented with sudden onset of severe radiating chest pain associated with diaphoresis, confusion, and loss of consciousness. Computed tomography (CT) revealed a dilated ascending aorta with a dissection flap in the arch and descending thoracic aorta. On transfer, the patient was awake and oriented with a blood pressure of 111/44 mm Hg and a heart rate of 101 beats/min. Cardiovascular examination was remarkable for bilaterally diminished radial pulses (2/4), and a 12-lead electrocardiogram showed sinus bradycardia with first-degree atrioventricular block. Multidetector electrocardiography-gated CT arteriography (CTA) revealed a 6.1-cm mid ascending aortic aneurysm. Proximally, a circumferential dissection flap at the aortic root extended above the coronary artery ostia. Distally, another flap in the aortic arch involved all 3 arch branch vessels. The dissection extended through the descending and abdominal aorta. Intraoperative transesophageal echocardiography (TEE) displayed a bicuspid aortic valve and moderately severe (3+) aortic valve regurgitation with a central jet.

The extensive disease necessitated complete resection of the aortic arch and proximal descending aorta. The patient underwent aortic root reconstruction with a composite valve graft. The proximal descending thoracic aorta was reconstructed—folding the adventitia over the intima and sewing the layers together with running suture. A 24-mm Hemashield graft (Getinge) was sewn to the proximal descending thoracic aorta, and the head vessels were sewn to the side of the graft. A 32-mm Hemashield graft was sewn at the level of the sinutubular junction and distally to the repaired aortic arch. The patient was taken to the intensive care unit in stable condition and discharged home on postoperative day 11. At 6 months, the patient showed a stable proximal repair and healed arch with a residual descending AD.

### Patient 2

**Figure 2:**
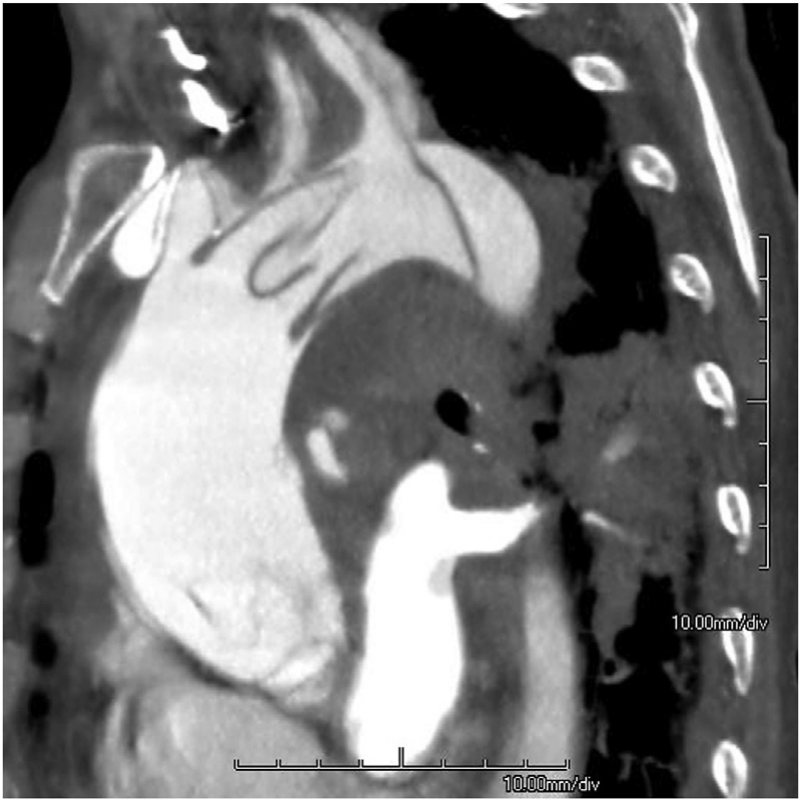
A sagittal section of a dilated mid ascending aorta with a proximal dissection flap in the aortic root and a distal flap in the aortic arch.

A 49-year-old woman with a past medical history of hypertension and obesity presented to the emergency department with severe, sharp, radiating chest pain coupled with confusion. Cardiovascular examination was remarkable for distant heart sounds along with hypotension and tachycardia. Electrocardiography revealed ST-segment elevation in aVR and ST segment depression in V_1_. Emergent CTA revealed a Stanford type A AD extending from the ascending aorta to the iliac arteries. A proximal circumferential dissection flap was difficult to detect on CT but confirmed on direct visualization. A distal circumferential dissection involving the proximal great arteries was clearly noted (Figure 2). Intraoperative TEE revealed severe aortic regurgitation (4+), and transection of the aorta showed a circumferential tear in the mid ascending aorta. Intussusception of the proximal flap extended across the LVOT, obstructing flow into both coronary arteries. Distal intussusception carried the intima into the aortic arch. A 26-mm Hemashield graft with a 10-mm side limb was beveled and sewn to the underside of the aortic arch, and reconstruction was performed. Hemiarch fixes can bring the circumferential intima into the ascending aorta, thus allowing re-creation of aortic layers. The patient was discharged on postoperative day 16. At 3 months, she showed a stable proximal repair and no permanent brain damage.

### Patient 3

A 61-year-old woman with a past medical history of giant cell arteritis presented with tightness in her throat. Electrocardiography demonstrated minimal ST-segment depression and inverted T waves in leads V_5_ and V_6_. The chest radiograph was unremarkable, and she denied chest pain. The next morning, she was found unconscious, with a systolic blood pressure of 59 mm Hg and a heart rate of 65 beats/min. She was unresponsive to painful stimuli, although both pupils were reactive to light. Cardiovascular examination was remarkable for absent distal peripheral pulses. CT of the chest demonstrated a complex AD from the aortic root to the renal arteries, involving both carotid arteries. CT scan of the head revealed multiple lacunar and cerebellar infarcts. Intraoperative TEE showed a 5.0-cm dilated aortic root. The right ventricle was akinetic, and transection of the ascending aorta revealed a circumferential tear of the aortic intima, contained only by visceral tissue between the aorta and pulmonary artery. Intussusception distally involved the aortic arch, with occlusion of the head vessels extending to the right axillary artery. Proximally, intussusception extended to the base of the noncoronary cusp, with occlusion of the right coronary artery and severe aortic insufficiency. The aortic valve was replaced, along with a hemiarch tubular graft extending into the proximal arch repair. Although hemodynamically improved postoperatively, the patient remained neurologically unresponsive and progressed to brain death. Pathologic examination of aortic specimens was consistent with giant cell arteritis.

## Comment

Stanford type A AD involves the ascending aorta and aortic arch and may extend to the descending aorta, carrying a death mortality rate of 60% within the first 24 hours and 90% within 3 months.^2^ Most AD is manifested with transverse intimal tears, rarely exceeding more than half of the aorta.^3^, ^4^, ^5^ Few instances of bidirectional intimo-intimal intussusception in the setting of acute type A AD have been reported, and such tears have significant sequelae, including obstructions of the LVOT and the aortic branch vessels. Although Nishioka and colleagues^6^ reported an isolated case of bidirectional AoII, this is a case series and discussion of combined proximal and distal circumferential AoII (Figure 1).

Circumferential tears are often manifested with distal/retrograde intussusception obstructing the aortic branch vessels.^2^ Distal intussusception into the aortic arch can lead to intermittent or permanent neurologic disturbances, as seen in patient 3. All 3 patients presented with a syncopal episode, and patients 1 and 2 regained consciousness after being hemodynamically stabilized, along with a complete recovery after surgical repair. Sequelae from a circumferential tear in the descending aorta may result in features suggestive of pseudocoarctation, with asymmetric pulses and blood pressure.^5^ Circumferential tears with proximal intussusception into the LVOT can occlude the coronary ostia and may cause myocardial ischemia. Such tears are also associated with acute aortic regurgitation due to intussusception through the valve orifice, as with patient 3.^1^^,^^3^

TEE is preferred in the diagnosis of AD because the lack of an intimal flap in the ascending aorta can be a source of false-negative errors in CT scans, especially in the axial plane.^4^^,^^5^ Proximal circumferential tears are better visualized on TEE, and the distal ascending aorta and aortic arch should be explored for a similar pattern of tears when indicated. Dissection in the aortic arch may be manifested as an unusual echocardiographic finding. Therefore, we recommend performing electrocardiography-gated CTA rather than simple CTA for better delineation of aortic arch anatomy.

Once diagnosed, patients with Stanford type A AD should undergo urgent surgical repair. Delay in treatment further complicates the pathologic process and increases the risk for serious morbidity. Operative strategies for AoII are consistent with those of usual AD—reduction and repair or replacement of the dissected segments of the aorta.^2^^,^^5^
